# An Improved Convolutional Neural Network Algorithm and Its Application in Multilabel Image Labeling

**DOI:** 10.1155/2019/2060796

**Published:** 2019-07-04

**Authors:** Jianfang Cao, Chenyan Wu, Lichao Chen, Hongyan Cui, Guoqing Feng

**Affiliations:** ^1^Department of Computer Science and Technology, Xinzhou Teachers University, Xinzhou 034000, China; ^2^School of Computer Science and Technology, Taiyuan University of Science and Technology, Taiyuan 030024, China

## Abstract

In today's society, image resources are everywhere, and the number of available images can be overwhelming. Determining how to rapidly and effectively query, retrieve, and organize image information has become a popular research topic, and automatic image annotation is the key to text-based image retrieval. If the semantic images with annotations are not balanced among the training samples, the low-frequency labeling accuracy can be poor. In this study, a dual-channel convolution neural network (DCCNN) was designed to improve the accuracy of automatic labeling. The model integrates two convolutional neural network (CNN) channels with different structures. One channel is used for training based on the low-frequency samples and increases the proportion of low-frequency samples in the model, and the other is used for training based on all training sets. In the labeling process, the outputs of the two channels are fused to obtain a labeling decision. We verified the proposed model on the Caltech-256, Pascal VOC 2007, and Pascal VOC 2012 standard datasets. On the Pascal VOC 2012 dataset, the proposed DCCNN model achieves an overall labeling accuracy of up to 93.4% after 100 training iterations: 8.9% higher than the CNN and 15% higher than the traditional method. A similar accuracy can be achieved by the CNN only after 2,500 training iterations. On the 50,000-image dataset from Caltech-256 and Pascal VOC 2012, the performance of the DCCNN is relatively stable; it achieves an average labeling accuracy above 93%. In contrast, the CNN reaches an accuracy of only 91% even after extended training. Furthermore, the proposed DCCNN achieves a labeling accuracy for low-frequency words approximately 10% higher than that of the CNN, which further verifies the reliability of the proposed model in this study.

## 1. Introduction

With the rapid development and increasing popularity of multimedia devices and network technologies, increasing amounts of information are being presented in image form. The large number of rich image resources has attracted users, who can find the information that they need in the images. According to statistics from Flickr, a website for social image sharing on the Internet, image storage is growing at an annual rate of 100 million units, while Facebook image storage is growing at a rate of 15 billion units per year [1]. However, this massive amount of image information can easily overwhelm users. Determining how to rapidly and effectively query, retrieve, and organize image information has become a major problem that must be solved [2]. As a result, the field of image retrieval technology has emerged and received considerable attention. Specifically, image annotation can provide more search information than traditional methods and result in rapid retrieval of corresponding images. However, because images often contain complex and diverse semantic information, they are typically labeled with more than one label; thus, it is necessary to consider the case of multilabel annotation.

Generally, the methods for automatically labeling multilabel images can be divided into three main categories: generative models, discriminant models, and nearest neighbor models. Generative models can generate training data randomly, particularly when certain implicit parameters are given. These models construct the joint distribution probability of the visual features and the text semantic tags first and then calculate the posterior probability of each semantic feature of the known image with a Bayesian probabilistic model, which they use to complete the semantic annotation of the image [3]. Duygulu et al. [4] proposed a generative model called the translation model, which transforms the image semantic annotation process into a translation process by transforming visual image keywords into semantic keywords. Jeon et al. [5] proposed the cross-media relevance model (CMRM), which models images to perform image annotation by constructing the joint probability of the visual and semantic information. Although the above model considers the semantics of objects and regions, the discrete processing of visual features can result in the feature loss. In addition, the labeling effect is largely influenced by the clustering granularity, but the ideal granularity parameters are difficult to determine in advance. To solve this problem, Feng et al. [6] proposed the multiple Bernoulli relevance model (MBRM), and Alkaoud et al. [7] proposed the fuzzy cross-media relevance model (FCRM). These models use a nonparametric Gaussian kernel to perform a continuous estimation of the feature generation probability. Compared with the discrete model, these models significantly improve the labeling accuracy. Although the annotation process of the abovementioned production annotation model is relatively simple, the gap between the underlying features of the image and the high-level semantics and the nonindependence among the semantics can lead to inaccurate joint probabilities [8].

A discriminative model defines image annotation as a traditional supervised classification problem. This approach performs image annotation mainly by determining the correlations between visual features and predefined labels [9]. The authors of [10] used the *K*-nearest neighbor (KNN) method to select the nearest *K* images by calculating the distance between graphs and then labeling the unlabeled image using a label propagation algorithm. Li et al. [11] used a *K*-means algorithm to construct a classifier by combining a semantic vocabulary with annotated words using semantic constraints and used the classifier for subsequent image annotation. Qiu et al. [12] used a support vector machine (SVM) to semantically denote some areas and then label unlabeled areas based on the relationships among the areas. Regardless of whether a method is based on one-to-one classification or one-to-many classification, it is subject to the constraints of the number of classifiers and the training effect of the classifier, especially in the case of unbalanced training samples. If the classifier training effect is poor, the overall labeling accuracy rate will be affected. As the size of the label set increases, the required classifier size also increases, which increases the complexity of the labeling model; thus, some methods may not be applicable in big data environments [13].

The nearest neighbor model has become popular as the requirements of data training have expanded. The authors of [14] introduced the transmission mechanism of nearest neighbor labeling. In this approach, image annotation is treated as a retrieval problem. The nearest neighbor depends on the averages of several distances calculated from visual features, also known as the joint equal contribution (JEC). For a given image, a label is passed through a neighbor. Visual characteristics such as color and texture are used for comparison and testing, and feature selection regularization is performed based on label similarity. However, this approach does not increase the sparsity or improve the accuracy of labels in all cases. The TagProp model [15] is a new type of nearest neighbor model. It creates combined weights based on the existence or nonexistence of neighbor labels and achieves good results. The traditional methods described above have advanced the field of image annotation, but they require manual feature selection, which can result in information loss, poor annotation accuracy, and a low recall rate [16].

In recent years, as deep learning has received increasing attention, some scholars have begun to apply deep learning to computer vision tasks. In 2012, Hinton et al. used a multilayer convolutional neural network to classify images using the widely used large-scale ImageNet database [17] for image recognition and achieved exceptional recognition results [18]. Since then, a large number of studies have developed improved network structures and increased CNN performance. For example, Google's GoogLeNet network [19] won the championship in the 2014 large-scale image recognition competition. The Visual Computing Group of Microsoft Research Asia developed a computer vision system based on a deep convolutional neural network that—for the first time—surpassed humans in its ability to identify and classify objects in the ImageNet 1000 challenge [20]. Although deep learning models have performed well on image recognition and classification tasks, most studies have focused on the network itself or on improvements in single-label learning. Specifically, the task of image annotation for multilabel learning has been given little attention, particularly for unbalanced datasets. Currently, the approaches to solve the issue of dataset imbalance primarily focus on the composition of the datasets themselves. Briefly, a balance is achieved in the entire dataset by reducing the number of the types of images that are overrepresented in the dataset (downsampling) or increasing the number of the types of images that are underrepresented (upsampling). Despite their easy operation, these approaches require the image quantities in the dataset to reach a certain level and therefore do not possess wide applicability. Images types that rarely occur in daily life are difficult to even obtain, let alone to obtain in large numbers, and simple translation transformation sometimes fails to satisfy the requirement.

By combining deep learning and multilabel image annotation and targeting the problems of insufficient training and the poor annotation effects of underrepresented images due to data imbalance, in this study, we design a dual-channel convolutional neural network (DCCNN) and propose a new multilabel image annotation method. To increase the annotation accuracy, particularly that of low-frequency words, the proposed model is designed with two input channels and one output channel constructed by two six-layer convolutional neural networks that use different parameters. One of the two input channels is trained on the entire dataset, while the other is specially trained on the low-frequency portions of the dataset. These two channels are independent; the low-frequency datasets undergo training two times, thus increasing the training weights of low-frequency words in the integral dataset. During testing, the outputs from both input channels are combined to form a joint decision, thus achieving an optimal annotation effect.

The main contributions of this study are summarized as follows:

1. The combination of deep learning and multilabel image annotation solves the issues of the complex annotation process, poor annotation efficiency, the deficiencies in determining characteristics, and the “semantic gap” that affect traditional annotation methods.
2. We design a DCCNN model that fuses two different convolutional neural networks. Based on an understanding of the convolutional neural networks themselves as well as the experimental results, the parameters are adjusted, and a fusion ratio is set between the two subnetworks that result in a satisfactory performance. The designed model is aimed at solving the poor annotation effect problem that occurs on underrepresented image types in datasets due to insufficient training. Compared with other methods to address such problems, the method proposed in this study is both convenient and fast, and its application is not restricted by datasets.
3. Based on an understanding of the general process of multilabel image annotation, this study proposes a multilabel image annotation algorithm that employs the DCCNN. This algorithm contains training and annotation phases, and the inputs and outputs differ according to the different phases. During the training phase, the two branch models are trained independently. Then, in the testing phase, these branch models are fused so that they exert joint contributions to decision-making with regard to the final annotation results.

## 2. Methods

### 2.1. Convolutional Neural Network

The first convolutional neural network (CNN) was proposed by Hubel and Wiesel [21] in the 1960s through studies of neurons in monkey cortexes related to local sensitivity and direction selection. CNNs use weighted sharing, downsampling, and local connection techniques that greatly reduce the number of required parameters and the complexity of the neural network. CNNs have been compared to traditional methods of image feature extraction such as the Histogram of Oriented Gradient (HOG) and Scale-Invariant Feature Transform (SIFT) methods; however, CNNs can typically extract more abstract and comprehensive features. In addition, CNNs avoid the need for complex image preprocessing because they can use the original images directly as input.

CNNs are mainly composed of a convolutional layer, a pooled layer, and a fully connected layer. The convolutional layer is a key part of the CNN. The function of this layer is to extract features from input images or feature maps. Each convolutional layer can have multiple convolution kernels, which are used to obtain multiple feature maps. The convolution layer is calculated as follows [22]:

(1) $$\begin{array}{c} x_{j}^{l}=f\left(\sum_{i\in M_{j}}x_{i}^{l-1}*k_{ij}^{l}+b_{j}^{l}\right), \end{array}$$

where *x*_*i*_^*l*−1^ is the characteristic map of the output of the previous layer, *x*_*j*_^*l*^ is the output of the *i*th channel of the *j*th convolution layer, and *f*(·) is called the activation function. Here, *M*_*j*_ is a subset of the input feature maps used to calculate *u*_*j*_^*l*^, *k*_*ij*_^*l*^ is a convolution kernel, and *b*_*j*_^*l*^ is the corresponding offset.

**Figure 1:**
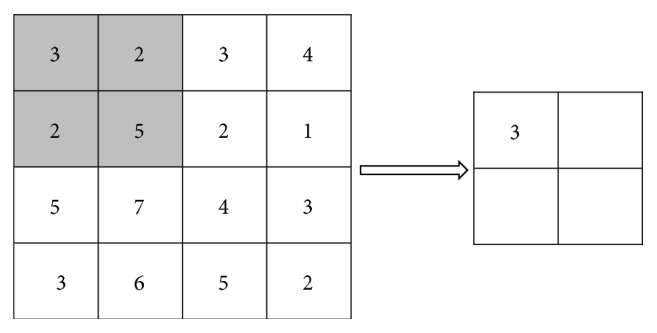
Pooling effect diagram. The left side of the diagram is a 4 × 4 feature map that consists of 2 × 2 pooling windows with a step length of 2 for downsampling the feature map. The right side shows the results after mean pooling, namely, the average value of the selected area is used as the pooled result. For max pooling, the pooled result would be the maximum value of the selected area.

A pooling layer is generally sandwiched between two convolutional layers. The main function of this layer is to reduce the dimensions of the feature map and maintain the scale invariance of the features to some extent. There are two main pooling methods: mean pooling and max pooling. A pooling effect diagram is shown in Figure 1.

The pooling process is similar to the convolution process in that it involves a sliding window similar to a filter, but the calculation is simpler. Mean pooling uses the average value in an image area as the pooled value of the area. This approach preserves the background of the image well. Max pooling takes the maximum value of the image area as the pooled value of the area and preserves the texture of the image well. The function of the fully connected layer is to integrate the multiple image maps obtained after the image is passed through multiple convolution layers and pooling layers to obtain the high-layer semantic features of the image for subsequent image classification.

### 2.2. Dual-Channel Convolutional Neural Network (DCCNN)

**Figure 2:**
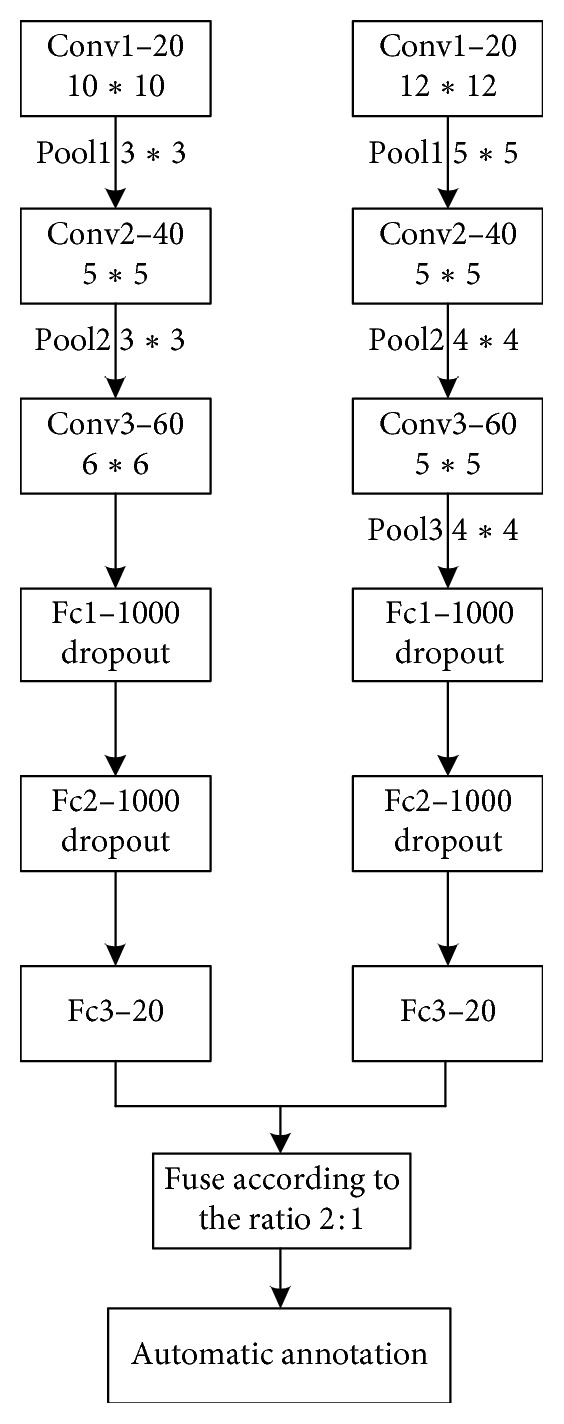
Structure of a two-channel convolutional neural network. Conv = convolutional layer; Pool = pooling layer; Fc = fully connected layer. The *x* in conv-*x* denotes the number of convolution kernels in the layer; the size of the convolution kernel or pooling window in the layer is *y* × *y*; the *z* in Fc-*z* denotes the number of neurons in the fully connected layer; and dropout shows the addition of a dropout layer to the original layer.

In the image annotation problem, one image often corresponds to a plurality of annotated words, and different annotated words correspond to different scenes. Some scenes correspond to many images, that is, the corresponding frequency of the annotated words is large, such as sun, white clouds, mountains, and rivers. Additionally, some scenes correspond to few images, and their corresponding word frequency is small, such as crocodile and lizard. Unbalanced input data can result in the insufficient training of low-frequency annotated words, resulting in a poor recognition rate. To increase the recognition accuracy and the overall recognition efficiency for low-frequency annotated words, this paper designs a DCCNN model (Figure 2).

In Figure 2, the DCCNN model consists of the convolutional neural networks CNN0 and CNN1. Each of these networks possesses three convolution layers and three fully connected layers.

CNN0 is trained on all training sets and the parameters of each layer are as follows:

  Layer 1 consists of 20 10 × 10 convolution kernels that perform convolution operations on the input images. The step length is initially set to 4. Then, 3 × 3 max pooling windows with a step length of 2 are used for downsampling.
  Layer 2 consists of 40 5 × 5 convolution kernels that perform convolution operations on the feature maps. The step length is initially set to 2. Then, 3 × 3 max pooling windows with a step length of 2 are used for downsampling.
  Layer 3 consists of 60 3 × 3 convolution kernels that perform convolution operations on the input feature maps. The step length is set to 1. The remaining three layers are fully connected layers. A dropout layer is applied for full connection to avoid overfitting. The keep pro (proportion) parameter is set to 0.5 (i.e., 50% of the neurons at each of the fully connected layers participate in the operation). The number of output nodes is 20. Considering that the ReLU activation function possesses powerful expression ability and is free from the vanishing gradient problem, enabling the convergence rate of the model to be maintained stably, we used the ReLU function in this study for all activations. The learning rate was adjusted to 0.001 after the experiment.

CNN1 is trained on the low-frequency training sets, and the parameters for each layer are as follows:

  Layer 1 consists of 20 12 × 12 convolution kernels that perform convolution operations on the input images. The step length is initially set to 2. Then, 5 × 5 max pooling windows with a step length of 4 are used for downsampling.
  Layer 2 consists of 40 5 × 5 convolution kernels that perform convolution operations on the input feature maps. The step length is initially set to 1. Then, 4 × 4 max pooling windows with a step length of 2 are used for downsampling.
  Layer 3 consists of 60 4 × 4 convolution kernels that perform convolution operations on the input feature maps. The step length is set to 1. Then, 4 × 4 max pooling windows with a step length of 2 are used for downsampling.
  The final three layers are all fully connected layers whose parameters are the same as those of CNN0.

The model increases the training weight of the low-frequency samples using special training channels. The training samples are first processed separately to achieve sample equalization. Then, during the labeling process, the final labeling result is jointly determined by the two channels. Because the low-frequency channel is trained only with low-frequency samples, the parameters of this channel are more suitable for identifying low-frequency samples, which reduces the labeling impact of training with insufficient numbers of low-frequency samples.

### 2.3. Multilabel Image Annotation

**Figure 3:**
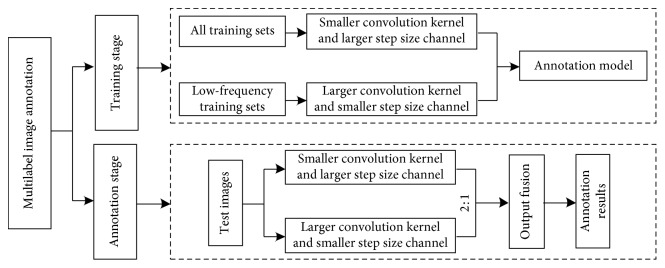
Multilabel image annotation framework.

In this paper, the labeling algorithm is divided into two phases: the training phase and the labeling phase, as shown in Figure 3.

The algorithm corresponding to the training phase is as follows.

 
*Step 1*. Launch the algorithm corresponding to the training phase. Sum the number of samples corresponding to each tagged word and determine the low-frequency annotation word set.
 
*Step 2*. Through the program, all the low-frequency samples in the training sample are extracted to form a low-frequency training set.
 
*Step 3*. Construct a CNN model with two channels: CNN0 and CNN1. CNN0 corresponds to the channel with a small convolutional kernel and a large step, and CNN1 corresponds to the channel with a large convolutional kernel and a small step. The first layer of the CNN1 channel is fully connected to the second layer of the fully connected layer for feature fusion.
 
*Step 4*. Input all the training sets into the CNN0 channel and input only the low-frequency training samples into the CNN1 channel. Conduct model training until the model becomes stable.

The labeling phase algorithm is as follows.

 
*Step 1*. Input the test image into both channels (CNN0 and CNN1) of the trained two-channel CNN for feature extraction
 
*Step 2*. Fuse the output vectors of the two channels in a 2 : 1 manner (the specific ratio is experimentally determined)
 
*Step 3*. Combine the decision results of the two channels to perform image annotation

### 2.4. Experimental Data

**Figure 4:**
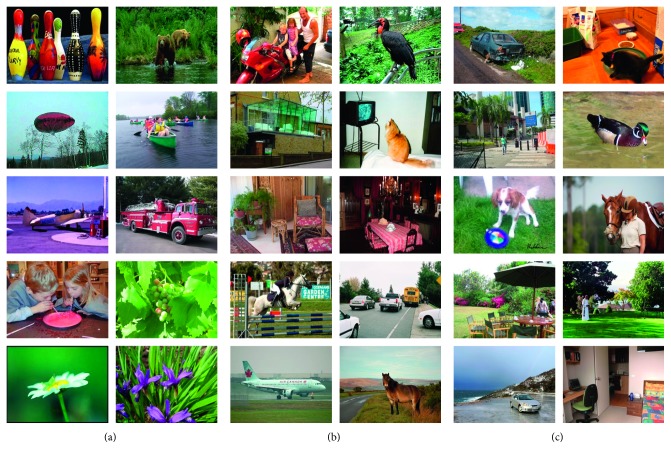
Example images from the Caltech-256 (a), Pascal VOC 2007 (b), and Pascal VOC 2012 (c) datasets.

To validate the proposed dual-channel CNN (DCCNN) algorithm, we performed experiments using the following freely available datasets: Caltech-256 [23], Pascal VOC 2007 [24], and Pascal VOC 2012 [25]. The Caltech-256 dataset contains 256 categories, each with at least 80 images and 30,608 overall images. The Pascal VOC 2007 dataset contains 20 categories and 9,963 images, with approximately 450 images per category. Based on the Pascal VOC 2007 dataset, Pascal VOC 2012 includes more images in each category, extending the dataset to 22,531 images over 20 categories, with each category containing approximately 1,000 images.  Figure 4 shows some example images from the three datasets.

### 2.5. Experimental Design

In this paper, we conducted simulation experiments based on the framework of the deep learning algorithm TensorFlow. Most of the images investigated in the multilabel image annotation task correspond to more than one label; thus, the evaluation criteria for single-label image classification are not fully applicable to multilabel image tasks. In this paper, we use mean average precision (MAP) as a new metric for multilabel images. The MAP score is obtained by calculating the average precision (AP). For a given task or category, the corresponding precision-recall curve can be calculated. Then, a set of thresholds is established [0, 0.1, 0.2,…, 1]. If the recall rate is greater than each threshold, the corresponding maximum precision value is obtained. AP is the average of the 11 precision scores, and its formula is as follows:

(2) $$\begin{array}{c} \text{AP}=\frac{1}{11}\sum_{r\in \left\{0,0.1,\ldots ,1\right\}}\text{Pinterp}\left(r\right), \end{array}$$

where Pinterp(*r*) is the maximum precision value corresponding to each threshold:

(3) $$\begin{array}{c} \text{Pinterp}\left(r\right)=\max_{\tilde{r}\%:\tilde{r}\%\geq r}p\left(\tilde{r}\right), \end{array}$$

and $p\left(\tilde{r}\right)$ is the accuracy rate corresponding to each $\tilde{r}$. Finally, the MAP is calculated by

(4) $$\begin{array}{c} \text{MAP}=\frac{\sum_{q=1}^{Q}\text{AP}\left(q\right)}{Q}, \end{array}$$

where *Q* is the number of categories.

We adopted MAP as a metric because it encompasses the accuracy, recall rate, and single-point value limitations of the *F*1 value and reflects the overall global performance.

Therefore, to verify the effectiveness of the proposed dual-channel CNN algorithm, we combine the characteristics of the three datasets (the Pascal VOC dataset image categories are small, and each category contains more images; the Caltech-256 dataset image categories are large, and each category contains fewer images) and compare the results with those reported in the literature in terms of labeling accuracy, automatic labeling effect, iteration number, and MAP score.

## 3. Results and Discussion

### 3.1. Labeling Accuracy Comparison

#### 3.1.1. Comparison on the Pascal VOC 2012 Datasets

The Pascal VOC 2012 datasets feature fewer categories and more images per category than does the Caltech-256 dataset. Therefore, we conducted an experimental comparison of the accuracy of the proposed DCCNN model with various methods from the literature [26–28] and the common CNN based on each category of the Pascal VOC 2012 dataset. The results are shown in Table 1.

Table 1 shows that the DCCNN method proposed in this paper yields a significantly higher labeling accuracy for each category than do the other four methods. Compared with the three methods from the literature and the CNN, the proposed method increased the MAP values on the Pascal VOC 2007 dataset by 42.7%, 13.1%, 16.7%, and 4.4%, and the MAP values on the Pascal VOC 2012 dataset increased by 48.4%, 17.1%, 18.8%, and 4.6%, respectively.

#### 3.1.2. Comparisons on the Mixed Datasets from Caltech-256 and Pascal VOC 2012

**Figure 5:**
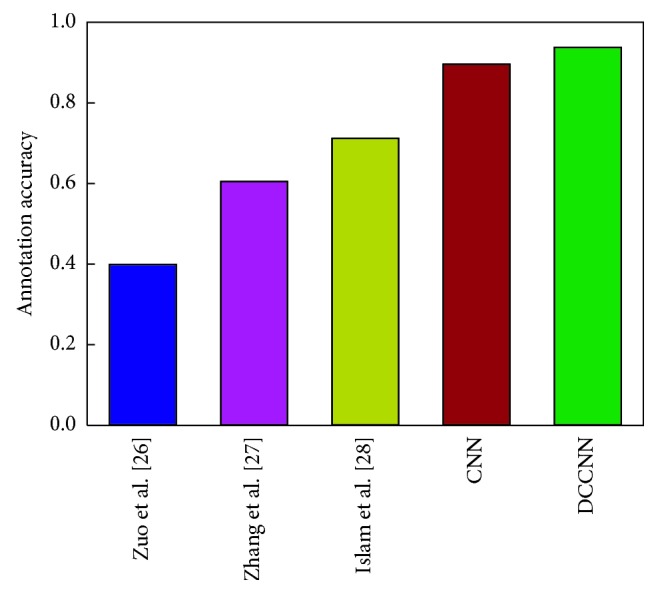
Comparison of the average labeling accuracy of different algorithms.

We combined the Caltech-256 and Pascal VOC 2012 datasets to form a larger dataset containing 50,000+ images and 276 categories and then compared the average labeling accuracy of the various models, as shown in Figure 5.

**Figure 6:**
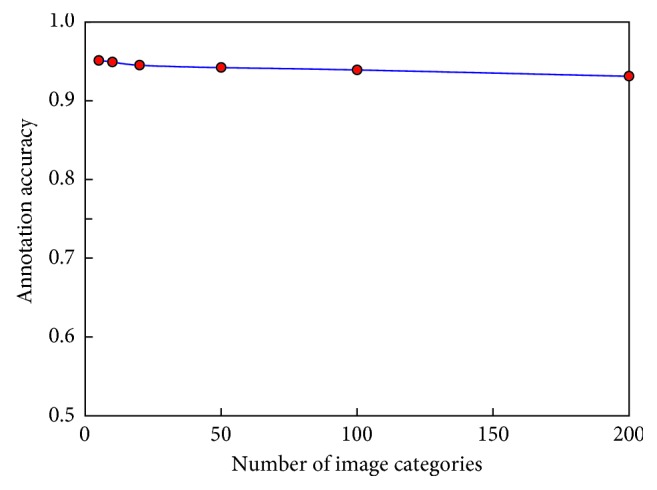
Relationship between the number of image categories and the labeling accuracy of the algorithm proposed in this study.

Additionally, to verify the influence of the number of image categories on the labeling accuracy of the algorithm proposed in this paper, we increased the number of image categories to evaluate the stability of the accuracy of the algorithm on the large dataset composed of Caltech-256 and Pascal VOC 2012 images. The results are shown in Figure 6.

The results in Figures 5 and 6 show that when the number of images reaches approximately 50,000, the DCCNN algorithm proposed in this paper achieves a higher labeling accuracy (93%) than do the other algorithms. Moreover, the results in Figure 6 show that the accuracy of the DCCNN method does not fluctuate as the number of image categories increases, further demonstrating that the DCCNN method can effectively avoid the information limitations associated with manual feature selection.

### 3.2. Comprehensive Comparison with a CNN Method

#### 3.2.1. Comparison of the Pascal VOC 2012 Datasets

To verify the improvement of the proposed method over the CNN algorithm, we first performed an experimental comparison of the accuracy of the two methods based on the same number of iterations using the Pascal VOC 2012 dataset.  Table 2 shows the experimental results when both models were trained 100 times.

Table 2 shows that the DCCNN proposed in this paper achieves good results within 100 training steps. The ordinary CNN achieves nearly the same effect only after being trained approximately 2,500 times. This finding indicates that the DCCNN proposed in this study is more accurate and efficient than the traditional CNN. The time and number of iterations required to reach the same accuracy percentage are shown in Table 3.

#### 3.2.2. Comparison of the Mixed Caltech-256 and Pascal VOC 2012 Datasets

**Figure 7:**
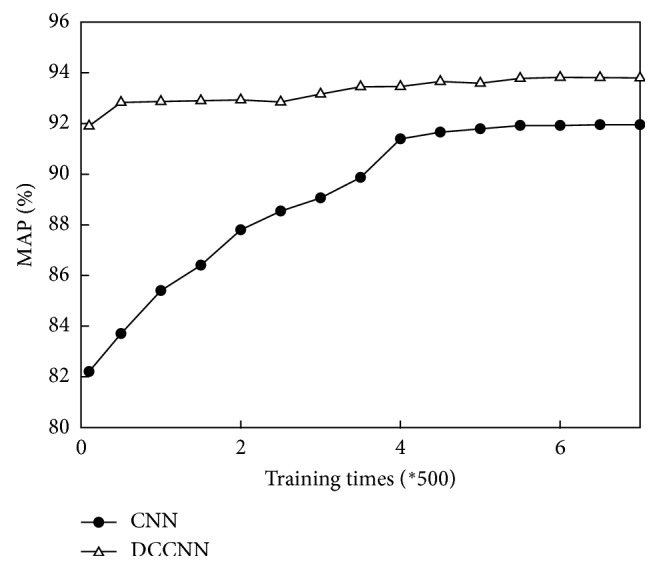
Comparison of the MAP values of the CNN and DCCNN.

For the large dataset consisting of Caltech-256 and Pascal VOC 2012 images, the MAP values of the CNN and the DCCNN are compared based on the number of training iterations required, as shown in Figure 7.

Figure 7 shows that the MAP value of the CNN is approximately 82% initially but finally stabilizes at 91.9%. In contrast, the MAP value of the DCCNN eventually reaches 93.8%, 1.9% higher than that of the CNN.

**Figure 8:**
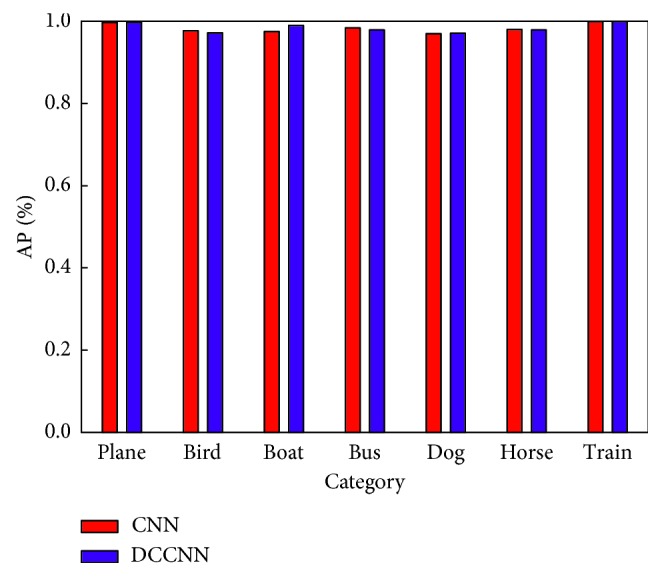
Comparisons of the AP values: CNN = convolution neural network; DCCNN = dual-channel convolution neural network.

In addition, we report the AP values of the two models for some vocabulary words after achieving stable training (Table 4 and Figure 8).

As shown in Table 4 and Figure 8, the annotation results of the CNN and DCCNN are similar because the effect of each model is nearly saturated after sufficient training. However, as shown in Table 3, the training rate of the CNN is much slower than that of the DCCNN. In addition, Figure 8 shows that the AP values of the DCCNN for some words are higher than those of the CNN, indicating a better annotation effect.

### 3.3. Comparisons of Low-Frequency Word Efficiency

To better verify the efficacy of the method proposed in this study, we compared the annotation effects of different methods on low-frequency words. As shown in Table 1, because the methods of [26–28] require manual feature selection, the labeling accuracy is very low for certain categories. According to [29], under the standard practice for low-frequency vocabulary, the highest and lowest frequency words are eliminated, and the frequencies of the remaining words are averaged. A labeled word corresponding to a below-average value is a low-frequency word. The results in Table 5 show that categories such as bike, bottle, chair, dining table, potted plant, sofa, and TV monitor are low-frequency words when labeling is based on manual feature selection.

#### 3.3.1. Comparison of Labeling Effects

In this section, we compare the DCCNN method with the method proposed in [27] and with CNN-based automatic labeling (Table 5).

The labeling results in Table 5 indicate that the DCCNN method provides more complete image descriptions and more comprehensive annotations than do the other two methods. Additionally, its recognition rate for low-frequency words such as chair, sofa, TV monitor, and dining table is higher.

#### 3.3.2. Comparison of Annotation Accuracy

**Figure 9:**
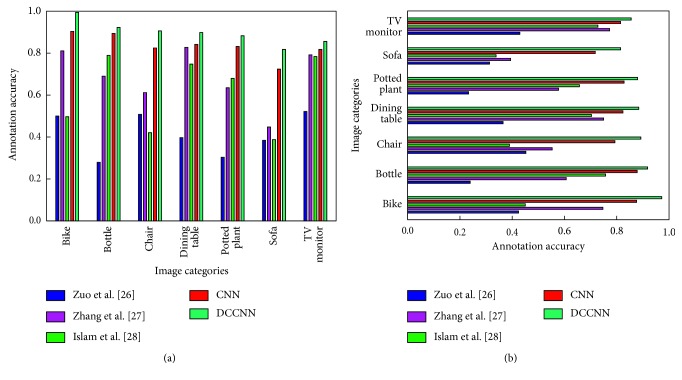
Comparison of the labeling accuracy for low-frequency vocabulary based on different algorithms: (a) accuracy based on the Pascal VOC 2007 dataset and (b) accuracy based on the Pascal VOC 2012 dataset.

For low-frequency words, as shown in Figure 9, the labeling accuracies when using the CNN and the DCCNN method proposed in this paper are significantly higher, and the DCCNN method yields the highest accuracy. This result indicates that compared with traditional methods (the methods in [26–28]), the extracted image features are more abstract, more comprehensive, and more suitable for the high-level semantics associated with the human understanding of images. Therefore, its labeling accuracy is significantly higher. The DCCNN model proposed in this paper is an integrated model of two different CNNs. One CNN has a small convolutional kernel and a large step, and the other has a large convolutional kernel and a small step. During training, to increase the training weights of low-frequency vocabulary words, images corresponding to the low-frequency vocabulary are input into the CNN with a large convolutional kernel and a small step. In contrast, the other CNN is trained on all the training images. This approach results in the DCCNN having a higher accuracy than the classical CNN method alone.

**Figure 10:**
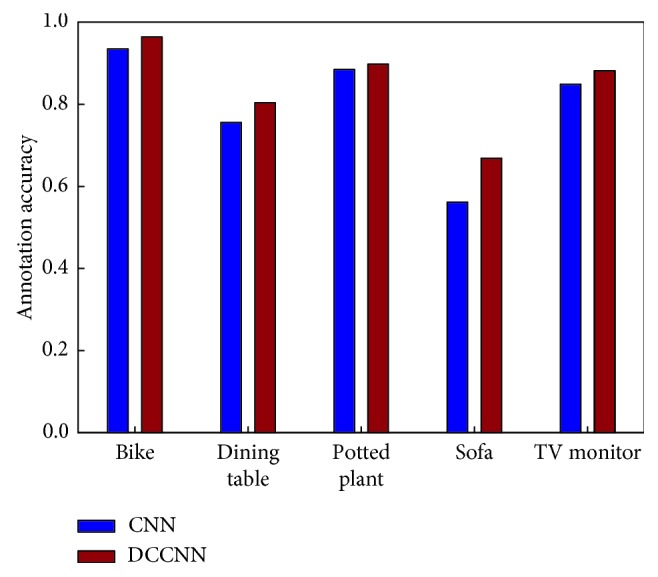
Comparison of the labeling accuracy for specific low-frequency terms.

Moreover, we also evaluated the annotation accuracy of each method based on some low-frequency words in the large dataset composed of Caltech-256 and Pascal VOC 2012 images after the two models became relatively stable. The experimental pairs are shown in Table 6 and Figure 10.

Figure 10 is a visual diagram of the information in Table 6. Most of the average accuracy improvements are similar. The annotation accuracies of “bike,” “dining table,” “potted plant,” “sofa,” and “TV monitor” increased by 2.9%, 4.8%, 1.3%, 10.7%, and 3.3%, respectively, when using the DCCNN rather than the CNN. Thus, the DCCNN designed in this paper improves the proportions of low-frequency annotated words in the model, further validating the effectiveness of the model.

## 4. Conclusions

In real life, with the continuous development of technology, access to information has grown explosively, and the amounts of image data have increased dramatically. Determining how to efficiently and rapidly solve large-scale image processing problems has become a popular and challenging research topic. Automatic image annotation is the key to text-based image retrieval. While the traditional methods made significant progress in the field of image annotation, their reliance on manual feature selection caused some information to be missed, resulting in poor annotation accuracy and low recall rates. Although deep learning models have been successfully applied for image recognition and classification, most studies have focused on the specific network used or on improving single-label learning. Notably, the application and improvement of multilabel image annotation in deep learning have been given little attention.

Therefore, based on the characteristics of multilabel learning and considering the uneven distribution of labeled words, we propose a DCCNN to improve the training weights of low-frequency words and the overall labeling efficiency. We validated the model with classic, commonly used multilabel image datasets: the Caltech-256, Pascal VOC 2007, and Pascal VOC 2012 datasets. In this study, we compared the DCCNN with existing methods from the literature and a traditional CNN. The methods based on CNNs are more effective for image annotation than are the traditional methods based on the manual feature selection. We also conducted a comprehensive comparison between the DCCNN and CNN. The results verify that the DCCNN improves both the accuracy of low-frequency vocabulary labeling and the overall labeling efficiency.

The next steps in this research are threefold: (1) training samples will be grouped according to the word frequency, and a multichannel CNN model will be established to further reduce the influence of word frequency on the model; (2) the labeling results will be further improved by considering the symbiotic relationships among words and the distances between mapped words; and (3) tests will be performed using larger datasets. Finally, based on the results, we will make improvements that further enhance the stability of the solution. The use of larger image datasets has certain benefits for network training and avoids overfitting.

## Figures and Tables

**Table 1:** Comparison of the accuracy of labeling for each category in the Pascal VOC 2007 and Pascal VOC 2012 datasets based on different algorithms.

Image category	Labeling accuracy									
	Zuo et al. [26]		Zhang et al. [27]		Islam et al. [28]		CNN		DCCNN	
	2007	2012	2007	2012	2007	2012	2007	2012	2007	2012
Plane	0.802	0.777	0.988	0.973	0.947	0.924	0.992	0.983	1.0	0.999
Bike	0.501	0.425	0.812	0.748	0.498	0.451	0.905	0.877	0.996	0.973
Bird	0.561	0.454	0.873	0.808	0.962	0.946	1.0	0.977	1.0	0.984
Boat	0.619	0.533	0.899	0.853	0.671	0.652	0.935	0.920	0.990	0.972
Bottle	0.28	0.24	0.691	0.608	0.791	0.758	0.895	0.879	0.924	0.919
Bus	0.784	0.722	0.931	0.899	0.966	0.951	0.976	0.971	0.986	0.980
Car	0.584	0.506	0.897	0.868	0.905	0.891	0.953	0.949	0.989	0.987
Cat	0.607	0.542	0.941	0.893	0.941	0.923	0.962	0.955	0.984	0.970
Chair	0.509	0.453	0.613	0.554	0.422	0.39	0.826	0.794	0.907	0.893
Cow	0.309	0.26	0.848	0.778	0.866	0.857	1.0	0.999	1.0	1.0
Dining table	0.398	0.366	0.829	0.751	0.749	0.704	0.843	0.825	0.899	0.885
Dog	0.507	0.426	0.885	0.83	0.895	0.886	0.918	0.905	0.992	0.971
Horse	0.441	0.389	0.916	0.875	0.912	0.894	0.929	0.927	0.980	0.978
Motorbike	0.57	0.507	0.825	0.792	0.798	0.761	0.851	0.849	0.931	0.931
Person	0.769	0.703	0.899	0.847	0.831	0.794	0.899	0.897	0.962	0.957
Potted plant	0.305	0.234	0.636	0.578	0.681	0.658	0.833	0.829	0.884	0.881
Sheep	0.406	0.362	0.824	0.792	0.895	0.862	0.960	0.960	0.996	0.993
Sofa	0.385	0.314	0.449	0.395	0.389	0.339	0.725	0.719	0.819	0.816
Train	0.699	0.616	0.935	0.906	0.864	0.818	1.0	0.989	1.0	1.0
TV monitor	0.523	0.43	0.793	0.774	0.785	0.729	0.819	0.816	0.857	0.856
MAP value	0.528	0.463	0.824	0.776	0.788	0.759	0.911	0.901	0.955	0.947

**Table 2:** Comparison of the labeling accuracy when two models were trained 100 times on the Pascal VOC 2012 dataset.

Image category	CNN	DCCNN
Plane	0.983	0.999
Bike	0.877	0.973
Bird	0.918	0.984
Boat	0.920	0.972
Bottle	0.722	0.892
Bus	0.920	0.980
Car	0.819	0.939
Cat	0.916	0.970
Chair	0.668	0.804
Cow	0.999	1.0
Dining table	0.570	0.757
Dog	0.894	0.971
Horse	0.927	0.978
Motorbike	0.849	0.931
Person	0.871	0.957
Potted plant	0.729	0.881
Sheep	0.960	0.993
Sofa	0.618	0.827
Train	1.0	0.999
TV monitor	0.746	0.866
MAP value	0.845	0.934

**Table 3:** Comparison of time and number of iterations required to reach a MAP value of 93% using the CNN and DCCNN.

Methods	CNN	DCCNN
Time (s)	277.411	41.446
Number of iterations	2500	100

**Table 4:** Comparisons of the AP values of the convolution neural network (CNN) and the dual-channel convolution neural network (DCCNN) to part of the vocabulary.

Vocabulary	CNN	DCCNN	Difference in AP
Plane	0.997	0.998	0.001
Bird	0.977	0.972	0.005
Boat	0.975	0.990	0.015
Bus	0.984	0.979	0.005
Dog	0.970	0.971	0.001
Horse	0.980	0.979	0.001
Train	1.0	0.999	0.001

**Table 5:** Comparison of the labeling results of different algorithms.

Sample image	Automatic labeling results		
	Islam et al. [28]	CNN	DCCNN
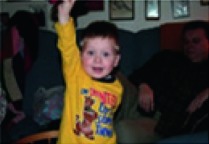	People	People, sofa	People, sofa, chair

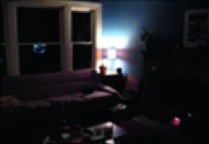	Sofa	Sofa, potted plant	Sofa, potted plant, chair, TV monitor

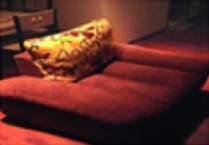	Chair, sofa	Chair, sofa	Chair, sofa, dining table

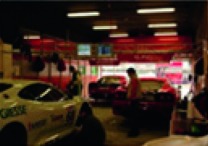	People, car	People, car	People, car, TV monitor

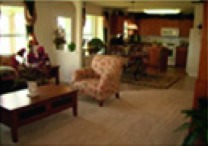	Chair, dining table	Potted plant, chair, dining table	Potted plant, chair, dining table, sofa

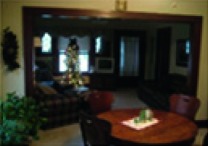	Dining table, chair	Dining table, chair, potted plant	Dining table, chair, potted plant, sofa

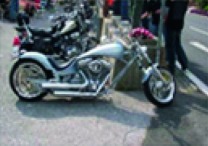	Motorbike	People, motorbike	People, motorbike, chair

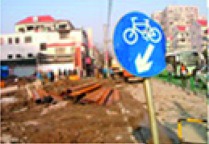	People, bus	People, bus, car	People, bus, car, bike

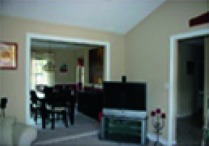	Chair, TV monitor	Sofa, chair, TV monitor	Sofa, chair, TV monitor, dining table

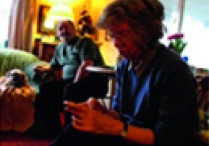	Chair, people	Dog, chair, people, sofa	Dog, chair, people, sofa, potted plant

**Table 6:** Comparison of the labeling accuracies of the CNN and DCCNN for low-frequency words.

Low-frequency words	Labeling accuracy		
	CNN	DCCNN	Difference
Bike	0.935	0.964	0.029
Dining table	0.756	0.804	0.048
Potted plant	0.885	0.898	0.013
Sofa	0.562	0.669	0.107
TV monitor	0.849	0.882	0.033

## Data Availability

The data used to support the findings of this study are included within the article.
